# Using fosfomycin to prevent infection following ureterorenoscopy in response to shortage of cephalosporins: a retrospective preliminary study

**DOI:** 10.1186/s12894-024-01530-8

**Published:** 2024-07-12

**Authors:** Toshiki Etani, Chiharu Wachino, Takuya Sakata, Maria Aoki, Masakazu Gonda, Nobuhiko Shimizu, Takashi Nagai, Rei Unno, Kazumi Taguchi, Taku Naiki, Shuzo Hamamoto, Atsushi Okada, Noriyasu Kawai, Atsushi Nakamura, Takahiro Yasui

**Affiliations:** 1 Department of Nephro-Urology, Graduate School of Medical Sciences, Nagoya City University, Kawasumi 1, Mizuho-Cho, Mizuho-Ku, Nagoya, Aichi 467-8601 Japan; 2 Division of Infection Prevention & Control, Nagoya City University Hospital, Kawasumi 1, Mizuho-Cho, Mizuho-Ku, Nagoya, Aichi 467-8601 Japan

**Keywords:** Cefotiam, Fosfomycin, Antibiotics, Ureteroscopy, Urolithiasis

## Abstract

**Background:**

In 2019, the shortage of cefazolin led to the demand for cefotiam and cefmetazole exceeding the supply. The Department of Nephro-urology at Nagoya City University Hospital used fosfomycin as a substitute for perioperative prophylaxis. This retrospective preliminary study evaluated the efficacy of fosfomycin and cefotiam for preventing infections following ureterorenoscopy.

**Methods:**

The study included 182 patients who underwent ureterorenoscopy between January 2018 and March 2021). Perioperative antibacterial treatment with fosfomycin (*n* = 108) or cefotiam (*n* = 74) was administered. We performed propensity score matching in both groups for age, sex, preoperative urinary catheter use, and preoperative antibiotic treatment.

**Results:**

The fosfomycin and cefotiam groups (*n* = 69 per group) exhibited no significant differences in terms of patients’ median age, operative duration, preoperative urine white blood cell count, preoperative urine bacterial count, and the rate of preoperative antibiotic treatment. In the fosfomycin and cefotiam groups, the median duration of postoperative hospital stay was 3 and 4 days, respectively; the median maximum postoperative temperature was 37.3 °C and 37.2 °C, respectively. The fosfomycin group had lower postoperative C-reactive protein levels and white blood cell count than the cefotiam group. However, the frequency of fever > 38 °C requiring additional antibiotic administration was similar.

**Conclusions:**

During cefotiam shortage, fosfomycin administration enabled surgeons to continue performing ureterorenoscopies without increasing the complication rate.

## Background

In 2019, a raw material shortage led to a cefazolin supply shortfall. In 2021, authorities penalized the manufacturer for production errors, further reducing the cefazolin supply. Japan designated cefotiam as a cefazolin alternative, but demand for cefotiam has exceeded the supply.

Ureterorenoscopy (URS) is a surgical procedure for crushing and removing kidney or ureteral stones using a rigid or flexible ureteroscope. Advancements in narrower ureteroscopes and lithotripsy equipment, such as the holmium yttrium–aluminium-garnet laser, have made URS the first choice in this context [1]. This approach is associated with complications in 9–25% of patients [2–4], with most complications being mild and resolving spontaneously. However, postoperative urosepsis may develop in ≤ 5% of patients [5, 6]. The Japanese Urological Association [7] advocates for the use of first- and second-generation cephalosporins, penicillins containing β-lactamase inhibitors, and aminoglycosides in URS. At Nagoya City University Hospital, cefotiam is used as prophylaxis during URS. However, the supply of cefotiam became unpredictable, rendering it unsuitable for prophylaxis during urological surgeries.

Therefore, following a discussion with the Division of Infection Prevention and Control at our institution, antimicrobials were used as alternatives to cefotiam and cefazolin for prophylaxis in patients requiring urologic surgery.

The first administration of fosfomycin was recorded in 1969 [8], and in recent years, its usefulness has been reconsidered owing to its effectiveness. Fosfomycin’s efficacy in urological surgery has been demonstrated in various procedures: 115 transurethral endoscopic surgeries, 20 clean surgeries, 54 clean-contaminated surgeries, and six contaminated surgeries [9]. Our previous research showed fosfomycin’s use in transurethral resection of bladder tumours [10] and endoscopic combined intrarenal surgery [11]. This study aimed to assess the effectiveness of fosfomycin sodium and cefotiam as prophylactic measures in URS.

## Methods

### Ethical consideration

This research adhered to the principles outlined in the Declaration of Helsinki. It was a retrospective analysis; therefore, informed consent from patients was not required (approved by the Ethics Committee of Nagoya City University Hospital). Information regarding withdrawal from the study was disseminated through the institutional website and bulletin boards. The protocol obtained approval from the Ethics Committee of Nagoya City University Hospital (approval number: 60–20-0033). Patient data is collected and analyzed without being taken outside the hospital. Electronic data is stored only on portable hard drives or in personal computers. The portable hard drives are stored in a locked cabinet, and the PCs are protected by passwords and wire locks.

### Study design/setting

The study included patients treated with URS at the Department of Nephro-urology, Nagoya City University Hospital (Nagoya, Japan) from January 2018 to March 2021. Patients received cefotiam (*n* = 74, Group 1) or fosfomycin (*n* = 108, Group 2) as perioperative prophylaxis within 1 h before URS to achieve adequate blood and tissue levels at the start of surgery, as recommended by Japanese guidelines for perioperative prophylactic antimicrobial administration [7]. Prophylactic prophylaxis therapy was routinely completed within 24 h following surgery. In Group 1, 1 g of cefotiam was administered once immediately before surgery, and 1 g every 12 h for additional doses. In Group 2, 2 g of fosfomycin was administered once immediately before surgery, and 2 g every 12 h for additional doses. Cefotiam was administered from January 2018 to April 2019, while fosfomycin was administered from May 2019 to March 2021. Ureterorenoscopy was performed by 17 surgeons using a Holmium YAG laser system, Cyber Ho 60 (EDAP TMS, Vaulx-en-Velin, France), as the lithotripter or haemostatic device. The study included all patients who underwent ureterorenoscopy during the specified period. Patients who declined to participate or received antimicrobial agents other than cefotiam or fosfomycin owing to drug allergy or other reasons were excluded.

### Outcomes and statistical analyses

Both groups had their maximum body temperature and postoperative hospital stay duration documented. Additionally, postoperative blood counts, blood biochemical tests, and preoperative and postoperative urinary sediment analyses were conducted. The preoperative urine analysis was performed on the day closest to the surgery, within a month’s timeframe. Postoperative blood analysis was conducted 24 h after surgery, and urinalysis was performed on an outpatient basis at 1 month. The analysis focused on the development of postoperative infections and complications. However, owing to the retrospective nature of this study, determining the development of such conditions in patients was challenging. Therefore, postoperative fever and additional antibiotic prescriptions were used as alternative endpoints.

Statistical significance was assessed using the Mann–Whitney U-test and the chi-squared test, performed with Statcel 4 software from OMS Publishing Company in Saitama, Japan. Statistically significant differences were indicated by *p*-values < 0.05. Propensity score matching were performed with EZR (Saitama Medical Center, Jichi Medical University, Saitama, Japan), which is a graphical user interface for R (The R Foundation for Statistical Computing, Vienna, Austria).

## Results

### Patients

Table 1 shows the characteristics of the 182 selected patients. The two groups exhibited similar values for several parameters, including patient sex, age, operative time, preoperative urine white blood cell (WBC) count, preoperative urine bacterial count, and the rate of preoperative antibiotic prescription. The groups were similar in terms of median operative time and median patient age. However, patients in the fosfomycin group were hospitalised for a shorter period than those in the cefotiam group. Preoperative antibiotic therapy was administered orally in all cases, with a median duration of 2 days. At the end of surgery, ureteral stents were placed in 95.9% of patients in the cefotiam group and 96.2% in the fosfomycin group. The ureteral stents were removed 3 weeks postoperatively using a cystoscope in an outpatient setting.

**Table 1:** Patient characteristics

Characteristic	Group1	Group2	*P*-value/Z value/Effect size
Patient number	74	108	-
Age (median, range; years)	60 (2–90)	65 (2–89)	0.68
Sex (n, %)			
Male	47 (63.6)	74 (68.6)	0.58
Female	27 (36.4)	34 (31.4)	
Preoperative urinary catheter (n, %)			
+	51 (68.9)	64 (59.2)	0.18
−	23 (31.1)	44 (40.8)	
Stone location (n, %)			0.79
R2	16 (21.6)	26 (24.0)	
R3	9 (12.2)	7 (6.5)	
U1	21 (28.4)	32 (29.6)	
U2	12 (16.2)	15 (13.9)	
U3	7 (9.5)	14 (13.0)	
Ureteroscopes other than lithotripsy	9 (12.1)	14 (13.0)	
Diameter of the stone (median, range; mm)	10.0 (3–24)	10.0 (3–38)	0.47
CT values of stones (median, range; HU)	1043 (219–1786)	1005 (308–2030)	0.28
Length of postoperative hospital stay (median, range; days) (confidence interval; days)	4 (2–11) (3.7–4.7)	3 (1–42) (3.2–4.9)	0.006*/2.42/0.06
Operative time (median, range; min)	72 (20–132)	75 (16–162)	0.09
Preoperative (4 weeks before surgery) blood test results			
WBC (median, range; /μL)	5,950 (3,600–15,700)	6,300 (3,100–13,400)	0.58
CRP (median, range; mg/dL)	0.08 (0.03–8.0)	0.10 (0.03–2.7)	0.44
Creatinine (median, range; mg/dL)	0.81 (0.06–4.5)	0.93 (0.2–4.5)	0.85
Total bilirubin (median, range; mg/dL)	0.6 (0.2–2.1)	0.7 (0.2–3.6)	0.65
AST (median, range; U/L)	20 (11–92)	21 (12–126)	0.74
ALT (median, range; U/L)	18 (4–134)	18.5 (8–123)	0.86
Preoperative urine WBC count (median, range; /μL)	46.6 (0.9–9564)	34.2 (0.1–8027)	0.08
Preoperative urine bacteria count (median, range; /μL)	44.8 (0.8–61,488)	50.1 (0.0–49603)	0.19
Preoperative antibiotic treatment (n, %)	3 (4.1)	5 (4.6)	0.85

Except for the duration of postoperative hospital stay, no significant differences were observed between the two groups in terms of the main parameters

*CTM* Cefotiam, *FOM* Fosfomycin, *WBC* White blood cells, *R2* the renal pelvis and renal calyx, *R3* the ureteropelvic junction, *U1* Upper ureter (with lower limit at the superior border of the iliac crest), *U2* Middle ureter (overlapping the iliac crest), *U3* Lower ureter (not overlapping the iliac crest). The range indicates the minimum and maximum values

^*^ *P* < 0.05 in the Mann–Whitney *U*-test

Propensity score matching was performed in both groups for age, sex, preoperative urinary catheter use, and preoperative antibiotic treatment. For the matched cohort analysis, 69 patients were selected from each group (Table 2). We analyzed these dependent variables using a logistic regression model. However, we could not use the caliper to analyze it since the original number of the control arm was relatively small, and we were afraid to lose a fair enough number of cases for the matching, weakening the statistical power. As a matter of fact, we could not confirm the sample size calculation due to the size of the case volume and the retrospective fashion of the dataset we had. In addition, any case that included any missing data regarding the dependent and independent variables was excluded from the matching.

**Table 2:** Patient characteristics (matched cohort analysis)

Characteristic	Group 1	Group 2	*P*-value/Z value/Effect size
Patient number	69	69	-
Age (median, range; years)	64 (48–74)	65 (64–71)	0.78
Sex (n, %)			
Male	47 (68.1)	46 (66.7)	0.85
Female	22 (31.9)	23 (33.3)	
Preoperative urinary catheter (n, %) Of these, patients with ureteral stent			
+	23 (33.3) 18 (26.1)	24 (34.8) 15 (21.7)	0.85
−	46 (66.7)	45 (65.2)	
Length of postoperative hospital stay (median, range; days) (confidence interval; days)	4 (3–5) (3.6–4.6)	3 (3–4) (3.0–5.4)	0.04*/1.95/0.03
Operative time (median, range; min)	72 (55–87)	75 (58–114)	0.07
Preoperative urine WBC count (median, range; /μL)	48.0 (8.7–180.0)	19.4 (6.6–79.1)	0.15
Preoperative urine bacteria count (median, range; /μL)	39.2 (8.1–752.0)	25.0 (10.3–152.0)	0.38
Preoperative antibiotic treatment (n, %)	3 (4.3)	5 (7.2)	0.71

No significant differences between the two groups were found in the primary parameters, except for the postoperative hospital stay duration

*CTM* Cefotiam, *FOM* Fosfomycin, *WBC* White blood cells. The range indicates the minimum and maximum values

^*^ *P* < 0.05 in the Mann–Whitney *U*-test

### Postoperative examination

Table 3 shows the findings of urine and blood testing. The groups exhibited similar results for postoperative urinary leukocyte and bacterial counts. Postoperative WBC counts and C-reactive protein concentration were lower in the fosfomycin group compared to the cefotiam group. Similar results were observed in both groups for maximum postoperative body temperature and concentration of creatinine, aspartate aminotransferase, alanine aminotransferase, and total bilirubin. Furthermore, the main stone components were consistent between the groups. The stone-free rate was 96.7% in the cefotiam group and 87.2% in the fosfomycin group (*P* = 0.07). Postoperative fever (> 38℃) requiring additional antibiotic treatment occurred in 10.1% and 12.1% of the patients in the fosfomycin and cefotiam groups, respectively. However, the treatment rates did not differ significantly.

**Table 3:** Postoperative examination data

Characteristic	Group 1	Group 2	*P*-value/Z value/Effect size
Postoperative urine WBC count (median, range; /μL)	25.6 (1.0–8191)	30.2 (0.1–13,765)	0.11
Postoperative urine bacteria count (median, range; /μL)	52.9 (0.0–49106)	47.0 (0.0–40466)	0.28
Postoperative (day 1) blood test results			
WBC (median, range; /μL) (confidence interval; /μL)	8,200 (3,100–24,400) (7924–8947)	7,500 (3,200–18,000) (7515–8682)	0.04*/2.02/0.19
CRP (median, range; mg/dL) (confidence interval; mg/dL)	0.7 (0.03–8.8) (1.0–2.0)	0.59 (0.03–14.5) (0.9–1.8)	0.009*/2.35/0.08
Creatinine (median, range; mg/dL)	0.85 (0.05–4.24)	0.89 (0.34–3.84)	0.57
Total bilirubin (median, range; mg/dL)	0.9 (0.4–2.7)	1.0 (0.2–2.7)	0.73
AST (median, range; U/L)	18 (9–72)	18 (9–52)	0.17
ALT (median, range; U/L)	16 (4–123)	17 (5–56)	0.37
Highest recorded body temperature (median, range; °C)	37.3 (36.4–40.0)	37.2 (36.4–39.0)	0.30
Postoperative fever > 38℃ requiring additional antibiotic treatment (n/%)	9/12.1	11/10.1	0.85
Component of stone (n, %)			
Calcium	58 (78.3)	80 (74.1)	0.54
MAP	2 (2.7)	1 (0.9)	
Others	1 (1.4)	1 (0.9)	
Not examined	13 (17.6)	26 (24.1)	

No significant between-group differences were observed in postoperative blood test results, except for WBC and CRP, maximum body temperature, and the rate of additional antibiotic treatment

*ALT* Alanine aminotransferase, *AST* Aspartate aminotransferase, *CRP* C-reactive protein, *CTM* Cefotiam, *FOM* Fosfomycin, *MAP* Magnesium ammonium phosphate, *WBC* White blood cells. The range indicates the minimum and maximum values

^*^ *P* < 0.05 in the Mann–Whitney *U*-test

Additional antibiotics administered postoperatively included fosfomycin, cefotiam, tazobactam/piperacillin, ampicillin/sulbactam, ceftazidime, ceftriaxone, and cefaclor. The daily doses were 2–4 g, 2–3 g, 13.5 g, 9 g, 2–4 g, 2 g, and 750 mg, respectively.

Surgical complications included ureteral injury in six patients in the fosfomycin group. In addition, ureteral stricture was already present at the time of surgery in three patients in the cefotiam group and eight patients in the fosfomycin group.

Table 4 demonstrates the results of the matched cohort analysis. The two groups yielded similar results with regard to blood testing and the incidence rate of postoperative fever > 38℃ that required further antibiotic administration.

**Table 4:** Postoperative examination data (matched cohort analysis)

Characteristic	Group 1	Group 2	*P*-value
Postoperative urine WBC count (median, range; /μL)	24.7 (8.4–106.7)	23.3 (6.3–127.4)	0.96
Postoperative urine bacteria count (median, range; /μL)	41.0 (7.6–221.4)	71.9 (15.2–356.5)	0.29
Postoperative (day 1) blood test results			
WBC (median, range; /μL)	8,200 (6,600–9,900)	7,700 (6,200–10,400)	0.64
CRP (median, range; mg/dL)	0.79 (0.44–2.07)	0.72 (0.24–1.49)	0.25
Creatinine (median, range; mg/dL)	0.87 (0.62–1.10)	0.89 (0.73–1.08)	0.38
Total bilirubin (median, range; mg/dL)	0.9 (0.6–1.2)	1.0 (0.8–1.3)	0.12
AST (median, range; U/L)	18 (15–22)	18 (15–21)	0.74
ALT (median, range; U/L)	16 (12–23)	16 (11–26)	0.96
Highest recorded body temperature (median, range; °C)	37.2 (37.0–37.6)	37.3 (37.1–37.7)	0.50
Postoperative fever > 38℃ requiring additional antibiotic treatment (n/%)	9/13.0	8/11.6	0.79
Component of stone (n, %)			
Calcium	54 (78.3)	46 (66.7)	0.27
MAP	2 (2.9)	1 (1.4)	
Others	1 (1.4)	1 (1.4)	
Not examined	12 (17.4)	21 (30.5)	

No significant between-group differences were observed in postoperative blood test results, except for WBC and CRP, maximum body temperature, and the rate of additional antibiotic treatment

*ALT* Alanine aminotransferase, *AST* Aspartate aminotransferase, *CRP* C-reactive protein, *CTM* Cefotiam, *FOM* Fosfomycin, *MAP* Magnesium ammonium phosphate, *WBC* White blood cells. The range indicates the minimum and maximum values

^*^ *P* < 0.05 in the Mann–Whitney *U*-test

### Urine culture

Preoperatively, urine cultures were conducted in 80.2% of the patients, with 40.1% yielding negative results. As shown in Table 5, at least two pathogens were detected in 13.2% of the patients. Gram-positive cocci (e.g., *Streptococcus* spp., *Enterococcus* spp*.*, *Staphylococcus* spp.) and *Escherichia coli* (*E. coli*) were the predominant microorganisms detected in this analysis (Table 6). Five methicillin-resistant strains of *Staphylococcus* spp. were identified. A total of 10 *E. coli* strains were detected, with only one being an extended spectrum β-lactamase-producing strain. Among *Staphylococcus* spp., 25% were susceptible to cefazolin and 87.5% to fosfomycin. Among *E. coli*, 90% were susceptible to cefazolin and 80% to fosfomycin. No significant difference was observed between the cefotiam and fosfomycin groups in the number or type of bacteria detected (chi-squared test, number of bacteria *p* = 0.31, species *p* = 0.14).

**Table 5:** Number of bacteria detected via preoperative urine culture

Number of detected bacteria or fungi	All patients (n/%)	Group 1 (n/%)	Group 2 (n/%)
0	73/40.1	25/33.8	48/44.4
1	49/26.9	18/24.3	31/28.7
2	17/9.4	4/5.4	13/12.0
3	6/3.3	4/5.4	2/1.8
7	1/0.5	0/0.0	1/0.9
Not examined	36/19.8	23/31.1	13/12.0

**Table 6:** Bacteria or fungi detected via preoperative urine culture

Detected bacteria or fungi	Total (n)	Group 1 (n)	Group 2 (n)
*Streptococcus* spp.	20	6	14
*Enterococcus* spp.	14	3	11
*Staphylococcus* spp.	12	3	9
Methicillin-resistant	5	2	3
*Escherichia coli*	10	7	3
ESBL-positive	1	1	0
*Corynebacterium* spp.	8	2	6
*Candida* spp.	6	2	4
*Proteus* spp.	4	3	1
*Lactobacillus* spp.	4	0	4
*Klebsiella* spp.	4	2	2
*Pseudomonas* spp.	3	1	2
*Gardnerella vaginalis*	2	1	1
*Bifidobacterium* spp*.*	2	1	1
*Actinotignum schaalii*	2	0	2
*Enterobacter* spp.	2	2	0
*Citrobacter* spp.	1	0	1
*Aerococcus urinae*	1	0	1
Negative	73	25	48

*ESBL* Extended-spectrum β-lactamase

## Discussion

The findings of this study revealed that during the shortage of cefazolin and cefotiam, fosfomycin was effective against post-URS infections.

Previous reports indicate that the incidence of post-URS infections ranges from 4–11.5% [12–14]. *E. coli* is frequently detected in patients experiencing postoperative bacteriuria following urologic operations. The occurrence of infections caused by other bacteria (e.g., *Staphylococcus aureus*) is also a concern during these procedures [13, 15–19]. In patients undergoing URS, a positive preoperative urine culture is a risk factor for postoperative sepsis. Therefore, administering prophylactic antimicrobial agents that are effective against bacteria detected in preoperative urine cultures is essential [20, 21].

According to the guidelines of the Japanese Urological Association, it is recommended to provide prophylaxis before URS by administering a single dose of first- or second-generation cephalosporins, β-lactamase inhibitor-containing penicillin, and aminoglycosides [7]. In our hospital, a single dose of prophylactic antimicrobials is currently administered during URS. However, in this slightly dated study, prophylactic doses were also administered within 24 h postoperatively.

In a randomised controlled trial, an investigation assessed the necessity of prophylactic antimicrobial therapy in URS. The study found that patients who received a single dose of levofloxacin had a significantly reduced incidence of postoperative bacteriuria compared to those who did not receive prophylactic antimicrobial therapy [22]. Another randomised controlled study compared the frequency of bacteriuria after surgery in patients who did and did not receive a single dose of cefazolin. Administration of cefazolin significantly reduced the incidence of bacteriuria (3.5% vs. 35%, respectively) [23]. A meta-analysis involving patients without preoperative urinary tract infections demonstrated that the administration of a single dose of antibiotics during surgery significantly reduced the rates of post-URS pyuria (relative risk: 0.65) and bacteriuria (relative risk: 0.26), as well as the incidence of febrile urinary tract infection. However, the difference did not achieve statistical significance [24]. Neither analysis identified any variation in the frequency of symptomatic urinary tract infections following the operation. In a separate study, Togo et al. reported a 4% occurrence rate of postoperative urinary tract infections after treating patients undergoing URS with a single dose of first- and second-generation cephalosporins and penicillins [12]. In a retrospective report that compared a single dose of antimicrobials to a 2-day regimen, no significant difference was observed in the incidence rate of postoperative febrile urinary tract infection [25]. Despite this, it is essential to emphasise the importance of administering a single dose of prophylactic antimicrobials in URS.

Few studies have examined the types of prophylactic antimicrobial agents used in URS. As the supply of antimicrobial agents may be complicated, efforts should be made to increase the number of candidate antimicrobial agents for administration. Numerous reports have highlighted the effectiveness of fosfomycin as a prophylactic agent in various urological procedures, including data from 195 surgeries, such as 115 transurethral endoscopic surgeries, 20 clean surgeries, 54 clean-contaminated surgeries, and six contaminated surgeries [9]. Additionally, fosfomycin has shown efficacy in procedures such as transurethral surgery for urolithiasis [26, 27], transurethral resection of bladder tumours [28], and prostate needle biopsy [29, 30]. Fosfomycin (FOM) was discovered in 1969 from Streptomyces fradiae isolated from soil sample cultures. It exhibits bactericidal activity against both Gram-positive and Gram-negative bacteria that cause postoperative infections, such as Escherichia coli and Staphylococcus aureus In vitro, fosfomycin is bactericidal against Gram-positive and negative bacteria. In particular, it showed excellent antibacterial activity against Pseudomonas aeruginosa, Proteus spp., Morganella morganii, Serratia marcescens, and multidrug-resistant Staphylococcus aureus and Escherichia coli. The mechanism of action of fosfomycin is quite unique. Fosfomycin is efficiently taken up into the bacteria by the active transport system of the cytoplasmic membrane and shows antibacterial activity by inhibiting the biosynthesis of cell wall peptidoglycans in the initial stage. Another advantage is that the unique structure of phosphomycin is less allergenic. In Japan, it is rarely used as a first choice, making it a useful option in terms of dispersing antimicrobial agents to prevent the development of resistance.

In the present research, the fosfomycin group did not exhibit worse outcomes than the cefotiam group in terms of blood test results, duration of hospital stay, and rate of additional antimicrobial administration. Currently, evidence regarding the use of prophylactic antimicrobials during URS is scarce, making the results of this study informative. Although the duration of hospitalisation was shorter in the fosfomycin group, this should not be interpreted as an indication of fosfomycin’s superiority over cefotiam. Since the fosfomycin group was studied later in the period, the shorter hospital stay reflects an effort to discharge patients as early as appropriate to manage hospital resources efficiently (in Japan, prolonged hospital stays can negatively impact hospital revenue). In addition, while postoperative WBC counts and C-reactive protein were significantly higher in Group 1, the matched cohort analysis showed no significant difference, suggesting no clear advantage between these two antibiotics regarding these parameters. Our hospital is a university hospital with a backup system including an anesthesiology department and intensive care unit, and we have traditionally excelled in the treatment of urinary tract stones, and we have treated many difficult stone cases. Therefore, although strict comparisons with the profile of patients treated throughout Japan are necessary, the fact that fosfomycin was considered useful in our URS patients suggests that it may be useful in other populations as well. We believe that multicenter retrospective and prospective studies at the national level are needed to test this hypothesis.

This study had some shortcomings. We performed propensity score matching in both groups for age, sex, preoperative urinary catheter use, and preoperative antibiotic treatment. However, sampling bias may exist owing to the retrospective nature of the analysis. The propensity score matching may undervalue the statistical power due to the lack of caliper inclusion for the analysis and retrospective estimation based on small case volume. Furthermore, the investigation involved a small number of patients; increasing the number of patients by extending the study period could result in a more robust retrospective study with a larger sample size. Because this is a preliminary retrospective observational study, the content of patient information in the medical record may vary from patient to patient and may not provide information that could affect outcomes. Another problem is that retesting of urine cultures after preoperative antibiotic administration was left to the discretion of the physician in charge and therefore was not performed in the majority of cases. In addition, the number of patients in each of the cefotiam and fosfomycin groups is different, and the number of patients is limited. Therefore, potential bias affecting outcomes may not have been eliminated. To improve this, the retrospective or prospective study should be conducted as a multicenter collaborative study with an even larger number of patients. Currently, the supply of cefazolin is stable, but difficulties in the supply of cefotiam are recurring. Therefore, increasing the number of patients included in the study is difficult because our hospital currently uses cefazolin as the prophylactic antimicrobial agent during URS. Prospective randomised trials comparing fosfomycin and cefotiam are warranted to examine the usefulness of fosfomycin as prophylaxis after URS. In addition, while urine cultures should have been tested preoperatively in all patients, only 80.2% of the patients in this study had urine cultures performed. Preoperative urine cultures yielded positive findings for half of the patients. Nevertheless, 4.6% and 4.1% in the fosfomycin and cefotiam groups, respectively, received preoperative antibiotic therapy.

Therefore, urine cultures should be performed before admission to the hospital, and urologists should be informed about the preoperative need for antimicrobial therapy based on the results of the cultures. It would also be beneficial to modify the electronic medical record system so that preoperative urine cultures are routinely performed. Furthermore, in patients who received preoperative antibiotic therapy, urine cultures should be retested to determine if bacteriuria has resolved after antibiotic treatment, which was not done in this study. Another limitation of this study is that the results of preoperative and postoperative urine cultures cannot be compared because routine postoperative urine cultures were not performed. Additionally, it would be desirable to evaluate various underlying diseases (such as diabetes, heart disease, renal disease, nutritional score, etc.) that may affect postoperative infectious and other complications. However, since this is a retrospective study relying only on medical records, this evaluation is insufficient. This point should be addressed in a prospective study.

## Conclusions

When a shortage of cefotiam occurs, the use of fosfomycin helps surgeons continue operations without increasing complication rates. The rise in resistant bacteria is a global problem, and the development of new antimicrobial drugs is slow. Existing antimicrobial agents that are not frequently used should be re-evaluated, and those found to be useful should be used proactively. Although fosfomycin is rarely used in daily urological practice in Japan, this study will help urologists realise its usefulness. Operations in which fosfomycin may be used include transurethral resection of bladder tumours, endoscopic combined intrarenal surgery, and URS. Future prospective investigations are necessary to evaluate the incidence of postoperative infections and compare fosfomycin with cefotiam.

## Data Availability

The data supporting the findings of this study can be obtained upon request from the corresponding author to protect patient privacy, as the data are not publicly available.

## Abbreviations

URS: Ureterorenoscopy
WBC: White blood cell
